# Traditional *Baduanjin* exercise through the eyes of patients with chronic heart failure: A qualitative content analysis study

**DOI:** 10.3389/fcvm.2022.1049036

**Published:** 2023-01-04

**Authors:** Xiankun Chen, Wei Jiang, Mariano Salazar, Huiying Zhu, Zehuai Wen, Xixi Chen, Cecilia Stålsby Lundborg

**Affiliations:** ^1^State Key Laboratory of Dampness Syndrome of Chinese Medicine, The Second Affiliated Hospital of Guangzhou University of Chinese Medicine, Guangzhou, China; ^2^Key Unit of Methodology in Clinical Research, Guangdong Provincial Hospital of Chinese Medicine, Guangzhou, China; ^3^Health Systems and Policy, Department of Global Public Health, Karolinska Institutet, Stockholm, Sweden; ^4^Department of Cardiology, Guangdong Provincial Hospital of Chinese Medicine, Guangzhou, China; ^5^Global and Sexual Health, Department of Global Public Health, Karolinska Institutet, Stockholm, Sweden; ^6^Science and Technology Innovation Center, Guangzhou University of Chinese Medicine, Guangzhou, China; ^7^School of Foreign Studies, Guangzhou University of Chinese Medicine, Guangzhou, China

**Keywords:** *Baduanjin*, chronic heart failure, experience, qualitative study, cardiac rehabilitation

## Abstract

**Objective:**

*Baduanjin* (eight silken movements) is a traditional Chinese exercise that can be used as cardiac rehabilitation therapy for patients with chronic heart failure (CHF) especially when other forms of rehabilitation are scarce or unaffordable. This study explores the experiences of Chinese patients with CHF who undertook *Baduanjin* exercise at home as part of a pilot trial in Guangzhou, China.

**Methods:**

We conducted seven qualitative interviews with participants who had participated in the intervention arm of a pilot randomized controlled trial (RCT) (*n* = 8). For data collection, we used a semi-structured interview guide with both open-ended, and follow-up questions. We audio recorded the interviews, transcribed them verbatim, and then analyzed them with content analysis.

**Results:**

Participants’ experiences of doing *Baduanjin* were classified into three categories: (1) improving practice (2) factors facilitating good exercise adherence, and (3) feeling good. Participants reported that the exercises were easy but that the correct *Baduanjin* execution and coordination between the mind, movements, and breathing were only achievable through practice. In addition, the training benefits which they perceived were the predominant motivation for patients to keep practicing. Finally, trust in *Baduanjin*, personal attitudes toward health, flexibility in practice times, as well as social support helped the participants to achieve good adherence to home-based training.

**Conclusion:**

This study’s findings indicate that *Baduanjin* could be a cardiac rehabilitation exercise modality for patients with CHF in China, especially in a home-based setting.

## 1. Introduction

Research findings have predicted that the prevalence of chronic heart failure (CHF) will continue to increase in the future on account of the aging population, risk factors’ increasing prevalence, and improvements in post-myocardial infarction survival. In China, heart failure mortality remains a significant public health concern, as the China Heart Failure Registry Study has reported an in-hospital mortality rate of 5.3% (1). CHF mortality rates are high, even for those patients who are compliant with the best available treatments (2). Studies have shown that exercise-based cardiac rehabilitation (EBCR) improves CHF patients’ exercise capacity and their quality of life (3, 4).

Despite its benefits, EBCR uptake remains sub-optimal around the world today (5). A key driver of this poor uptake is low availability due to insufficient resources such as rehabilitation facilities and trained professionals (6). A recent national survey in China has shown that only 24% of its tertiary hospitals have EBCR services (7). In addition, patient-related barriers have limited eligible patients’ participation in EBCR programs and/or regular exercise (8). The most common barrier is affordability (8), because EBCR services rely on out-of-pocket payment systems in China. There are other barriers to exercise as well, which are often related to practical issues such as timing, the convenience of travel to a rehabilitation center, or inclement weather (9).

A traditional form of exercise called *Baduanjin* can potentially contribute toward a solution to unmet demand for EBCR programs caused by their scarcity and unaffordability in China (7). *Baduanjin*, or “Eight Silken Movements” is a martial art that originated in ancient China, and has been practiced for over 1,000 years (10). It is characterized by interplay between flowing circular physical postures and movements, mindfulness, and breathing exercise which are conducted in synchronization (11). From a cultural perspective, it has been accepted as being beneficial to one’s health, and in modern times, it remains a popular exercise throughout Mainland China. Nowadays, with the proliferation of social media, *Baduanjin* exercise videos can easily be found online (12, 13).

The hallmark symptoms of CHF, such as fatigue and dyspnea, frequently cause decreases in physical activity, and erode the confidence necessary to initiate/maintain a regular exercise regimen (14). *Baduanjin*, however, has minimal physical or cognitive demands because it consists of only eight simple movements (10). Patients are also encouraged to do *Baduanjin* at home, reducing barriers such as weather, transportation, or cost (9). This equipment-free exercise might also be preferred by Chinese hospitals, especially those with limited resources in rural area (15).

*Baduanjin* has shown promise in cardiac rehabilitation in recent years (11, 16), especially among Chinese CHF patients (17–22). It has been shown to be a moderate-intensity aerobic exercise which is both an effective and a safe exercise modality for home-based cardiac rehabilitation for CHF patients (23). Results from a multi-center randomized controlled trial (RCT) have shown that 12 weeks of *Baduanjin* training can produce improvements in fatigue and quality of life (21). In addition, a growing body of evidence suggests that *Baduanjin* might be helpful to various CHF-related cardiac medical issues, including hyperlipidemia (24), fatigue (25, 26), hypertension (27), cardiorespiratory endurance (28), and that it also facilitates improvements in psychological health (29, 30).

Previous studies on *Baduanjin* efficacy have provided key quantitative data to assess its effectiveness (31); however, none of these studies have used qualitative methods to explore how patients experienced using *Baduanjin* in the context of cardiac rehabilitation and what are the factors facilitating or hindering its use. In addition, as a complex holistic “mind-body exercise” with influence from traditional Chinese medicine, *Baduanjin* may have additional health potential. Thus, information regarding the connection between *Baduanjin* and an individual’s subjective experiences may present new clinical outcomes.

With growing appreciation for patient-centered outcomes, it has been recognized that patient experience is equally as crucial as physiological tests and measurements for the successful implementation of intervention programs. Qualitative research, in which data is collected on participants’ personal experiences, is the ideal tool for exploring these complex experiences. This method may entail detailing experiences that are difficult to capture through quantitative research, and expanding our scientific understandings beyond what can be learned through quantitative analysis (32). Therefore, this study’s aim was to explore the experiences of Chinese CHF patients who use *Baduanjin* at home.

## 2. Materials and methods

### 2.1. Setting

This study was conducted in Guangzhou, China. Guangzhou is the capital of Guangdong province in Southeast China, and China’s third largest city. It has a permanent population of 13.5 million, and over 7 million permanent residents in its urban districts (33). Cardiac rehabilitation remains insufficiently implemented in current clinical practice in Guangzhou’s urban districts. Firstly, there is a low availability of cardiac rehabilitation services, especially in the outpatient clinics (7). Secondly, the practice patterns of cardiac rehabilitation are still very simple and poorly standardized (7). In most cases, cardiac rehabilitation comes in the form of verbal information supplemented by leaflets and booklets which emphasize early mobilization and identifying and controlling risk factors (34).

In addition, most existing programs are coordinated by a nurse, but relatively few used other healthcare professionals such as exercise physiologists, dieticians or physical therapists which is recommended in current guideline (34). Thirdly, there is no cardiac rehabilitation referral system after hospital discharge. Hence, there remain few cardiologists who regularly add cardiac rehabilitation to their daily clinical practice for their patients (7). Low physician referral may be compounded by the lack of hospital staff with training in cardiac rehabilitation resulting in inadequate knowledge about its benefits and inadequate assessment of a patient’s (in)ability to participate (7).

The participants of this study were taken from outpatient clinics at Guangdong Provincial Hospital of Chinese Medicine (GPHCM). GPHCM is a tertiary care public hospital with four campuses in different urban districts of Guangzhou. At GPHCM, cardiac rehabilitation is administered one-on-one by the cardiac nurses and rehabilitation physicians to individuals in a hospital outpatient clinic setting. It also includes exercise training. The most common exercise is cycle ergometer. However, this service is dependent on out-of-pocket payment systems. This creates financial stress for most patients, and in turn, results in low participation rates for cardiac rehabilitation.

To provide a way for heart failure patients to engage in a home-based EBCR program which is also equipment-free, low-cost, and easily implemented, we have developed a novel and contextually adapted EBCR program (BESMILE-HF) at GPHCM (35). BESMILE-HF is an acronym which stands for the Baduanjin Eight-Silken-Movement wIth SeLf-Efficacy building for Heart Failure. In this program, *Baduanjin* is applied as the core constituent in a multi-component EBCR which includes evaluation, consultancy, and education, in addition to a series of strategies for building self-efficacy. Our research group has conducted the pilot RCT to assess the feasibility and preliminary effects of *Baduanjin* in CHF patients. Pilot data suggested that *Baduanjin* may have a positive effect on clinical outcomes and may lead to increased long-term adherence to exercise through improved self-efficacy (36). As part of that project, this qualitative study was conducted.

### 2.2. Study design and participants selection

This qualitative study has been nested within a pilot RCT (36, 37). In the pilot RCT, participants were recruited from GPHCM. We assessed potential study participants for eligibility *via* (1) on-site patient screening during clinic visits; (2) regular screening of potential participants *via* the use of electronic medical records; and (3) physician referrals. To be included, patients had to have clinically stable CHF with a New York Heart Association functional (NYHA) classification of either II or III, and no restriction on the left ventricular ejection fraction class.

In the intervention group, the intervention was delivered by a cardiac rehabilitation team consisting of one cardiologist, a cardiology nurse, a professional coach, and research assistants. Before starting the 6-week home-exercise period, participants would attend an exercise course at the hospital to learn the eight postures, with a professional coach confirming their technique. This was followed by a 6-week phase consisting of home exercise with guidance and instruction from a *Baduanjin* exercise video, a picture-brochure, as well as weekly follow-up.

Generally, participants were required to do 30 min of *Baduanjin* per day, 5 days a week, for a total of 150 min each week. This was tailored to individuals based on their evaluation results. We asked the participants in the intervention group to record their exercise performance in exercise logs every day throughout the study period. The logs included duration in minutes and frequency. After 6 weeks, we contacted the participants and asked them to return to the hospital. Finally, there were 18 participants in the pilot RCT. After they signed informed consent, we randomized eight participants into an intervention group which received the 6-week BESMILE-HF program plus the usual medications, and 10 others into a control group which received only the usual medications.

Upon the conclusion of the pilot RCT, the eight participants from the intervention groups were invited to partake in semi-structured interviews. Seven participants agreed to be interviewed over 2 weeks. The ages of the seven interviewed participants spanned from 61 to 72 (mean = 67 years). All were male and married. Two participants had finished only primary school, one had finished secondary school, and four of the participants had college or university degrees. Participant characteristics are shown in Table 1. At the time of the interviews, the pilot RCTs’ outcome measure results were unknown.

**TABLE 1:** Characteristics of the interviewed participants.

ID	Sex	Age	Education level	NYHA class	LVEF sub-type	Exercise time ≥ 30 min/day?	Outcome of SEE
3	Male	67	College	II	HFrEF	Yes	Improved
6	Male	71	High school	III	HFpEF	Yes	Improved
8	Male	68	College	II	HFrEF	Yes	Improved
10	Male	62	Primary	III	HFpEF	Yes	Same
14	Male	72	College	II	HFpEF	Yes	Improved
15	Male	71	College	II	HFrEF	No	Improved
20	Male	61	Primary	III	HFrEF	Yes	Improved

NYHA, New York Heart Association functional; LVEF, left ventricular ejection fraction; HFrEF, heart failure with reduced ejection fraction; HFpEF, heart failure with perceived ejection fraction; SEE, self-efficacy for exercise (a questionnaire from pilot randomized controlled trial).

### 2.3. Data collection

Each participant was interviewed once using a semi-structured interview guide. A female research assistant (HZ) who is an attending physician with a background in cardiology was trained to conduct the interviews. She was not a member of the BESMILE-HF research team. All interviews were conducted face-to-face using Mandarin or Cantonese in the cardiac rehabilitation room at the hospital. On average, the interviews lasted around 40 min. The interview guide is based on a review of relevant literature (9, 38). It contains open-ended questions designed to gain a deeper understanding of the *Baduanjin* experience. We pilot tested the guide for clarity, relevance, and pertinence to the study’s goals and then the research team finalized it. We began with an introduction question: “Tell me about your experiences practicing *Baduanjin*?” Subsequent questions covered various aspects of *Baduanjin.* In the follow-up questions, participants were asked to elaborate. For the complete interview guide, refer to Table 2. HZ conducted the interviews, recorded them, and transcribed them verbatim, The transcripts were checked by XC (first author) by comparing the recording and the transcribed texts line-by-line.

**TABLE 2:** Interview guide.

Interview guide
1. Tell me about your experiences practicing *Baduanjin*.
2. How did you experience the exercise learning course?
-What did you think of the coach?
-Did you master the *Baduanjin* exercise after the course?
3. How do you practice *Baduanjin* at home?
-What you think of *Baduanjin* postures?
-What motivated you to practice? Or not to practice?
-Describe whether you have had any unpleasant experiences (like injuries or pain) or barriers when practicing *Baduanjin*.
4. What did you think of the effects on your daily life after practicing *Baduanjin*? Is it beneficial to you?
-If so, what benefits did you get?
-If not, what might be the reasons?
5. Do you have any suggestions regarding this exercise?

### 2.4. Data analysis

Two researchers (XC and HZ) performed qualitative content analysis based on an inductive approach utilizing the structure described by Elo and Kyngas (39) and assisted by NVivov11. Firstly, open coding was conducted. All transcribed texts were read several times with XC to make sense of the entire meaning, and then they were discussed for clarity with the interviewer (HZ). Then, the subjective experiences of *donging Baduanjin* were extracted. Next, we condensed the text into meaning units. Finally, the meaning units were coded. Examples of the resulting meaning units, condensed meaning units, and codes are shown in Table 3. Secondly, any codes with similar content were grouped into subcategories, and categories. Then, we sorted the subcategories and formulated them as categories. Co-author discussions were held throughout each step of the analysis until ultimately, there was consensus in terms of the structure of open coding and the structure of the sub-categories and categories. The analysis was initially conducted in Mandarin or Cantonese. Codes, sub-categories, and categories were then translated into English. The quotes presented are translated from Mandarin or Cantonese.

**TABLE 3:** An example of meaning units, condensed meaning units, and codes.

Meaning unit	Condensed meaning unit	Codes
I think my digestive system has become stronger and now I have a better appetite. Recently, I have been eating more than I did in the past	The digestion system has become stronger with a better appetite	Improved digestion
Moreover, I was often constipated before, but now I no longer suffer from constipation	The constipation has relived	Constipation relief

### 2.5. Ethical considerations

The BESMILE-HF trial has been approved by the Ethics Committee at the GPHCM (number: B2016-202-01) and also registered at ClinicalTrials.gov (NCT03180320). In addition, this qualitative study was also included in the trial protocol (35). Before each interview, we would ask the participant to read an information letter and provide both written and oral informed consent. We had an independent informed consent for the participants to take part in this qualitative study apart from the pilot study. Before giving consent, participants were allowed to ask questions and the personnel in the trial answered their questions carefully.

## 3. Results

Participants’ experiences of doing *Baduanjin* were classified into three categories (1) improving practice; (2) factors facilitating good exercise adherence; and (3) feeling good. Details of the codes, subcategories and categories are listed in Table 4.

**TABLE 4:** Categories, subcategories, and codes describing experiences with *Baduanjin* among chronic heart failure patients.

Categories	Subcategories	Codes
Improving practice	Postures are simple and easy to learn	Adequate intensity
		Suitable for the elderly
	Pursue postural accuracy	Mastering *Baduanjin’s* postures *via* video
		Importance of postural accuracy
		Challenges of reaching postural accuracy
	Realize the mind-body connection	Mastering *Baduanjin’s* inner essence over time
		Coordination of mind, body, and breath
		Awareness of their bodies and how their bodies felt
Factors facilitating good exercise adherence	Intrinsic motivation	General belief of improving general health
		Perceived training benefits
		One’s own choice
		One’s own care for the body
	External facilitator	Scheduling/routine
		Self-paced and equipment-free
		Exercise with friends/family members
		The exercise log and video is helpful.
Feeling good	Being active	More active in daily life
		Relieving dyspnea symptoms
		Increased confidence to exercise
	Physiological health benefits	Improved digestion
		Constipation relief
	Improved psychoemotional state	Feeling comfortable and relaxed
		Feeling calm, pleasurable, and anxiety-free
		Full of energy and vigor
		Better mood and hopeful
		Improved sleep quality

### 3.1. Improving practice

The category “Improving practice” provided insight into participants learning process. At the beginning of learning, participants found that *Baduanjin* was easy to learn and required little skill. In addition, participants perceived that its gentle and slow movements provided a suitable intensity level for the elderly. However, challenges for postures requiring them to squat, bend their waist, or turn their head were also reported.

*It only has eight movements! It is easy and I could master the postures after the course. You know, we have bad memories; it is hard for us to learn a new exercise. I think Baduanjin is simpler than other exercises. (ID: 6)*

As participants familiarized with the postures, they experienced the importance of reaching postural accuracy. They described that the maximum training effects were difficult to achieve in cases where the exercises had been done incorrectly.

*In the beginning, I had to follow the video to do the exercises. Slowly, I was able to remember it, but at the beginning, my movements were not very standard. Later, I found that I had to use force when doing the movements. This action has to be done in place, such as reaching the highest hand in the first posture and squatting in the fifth posture. Many people think that Baduanjin has no effect because they just do it casually without seriousness. Only when the movements are in place will they have an effect. (ID: 3)*

With *Baduanjin* practice over time, participants said that they had gradually mastered its inner essence. They described that practicing requires coordination of the mind, the body, and their breath. Only by coordinating breathing, body, and mind through training to harmonize by practicing *Baduanjin* can a practitioner achieve its benefits. In addition, participants identified that it was essential to be aware of their bodies and how they felt during practice in order to achieve a good practice.

*I have been doing it since 2004, and the effect of the previous years was not great, but the effect this time was much better. One reason was that I now coordinate my breathing. For example, in this movement, you must inhale when you stretch your arms backward, and exhale when you return them to the front, and breathe deeply. The other reason is the mind, that is to say, when you perform an action again, you have to know the purpose of the action. I used to just do exercises and not feel my body, so there was no effect. This time I learned that not only must I breathe well, but the idea of doing exercises is also very important. (ID: 3)*

### 3.2. Factors facilitating good exercise adherence

The category “Factors facilitating good exercise adherence” provide insight into the good adherence found in the pilot RCT. In the beginning, the reason they continued the exercises was the belief that *Baduanjin* can improve one’s general health. With the progression of *Baduanjin* training, participants emphasized that its perceived training benefits were the predominant motivation for them to adhere to it. Furthermore, participants reflected that “nobody can force me to exercise.” Whether or not to exercise was perceived as one’s own choice and this is determined primarily by the degree to which they cared for their own bodies.

*I do benefit from Baduanjin, or else I would not have participated for so long. (ID: 8)*

*I stopped because I did not feel any improvement*…*(ID: 15, non-adhering participant)*

In addition to those intrinsic motivations, several external facilitators were also reported to have supported participants’ sustained exercise. Participants told us that scheduling exercise or incorporating it into their routines promoted adherence. They also appreciated that the self-paced and equipment-free nature of the exercise allowed them to do it anytime and anywhere. They also reflected that they did not need a large training space or any other auxiliary apparatuses.

Practicing together with other friends or family members was found to facilitate practicing Baduanjin since it made it more enjoyable. Moreover, keeping exercise log was viewed as being helpful in keeping on track with their practice and reminding them to exercise daily. An exercise demonstration video was also a necessary tool to guide participants’ at-home exercise, especially at the outset when they were not so familiar with all eight postures.

*It is convenient for me to practice Baduanjin. I even recommended it to my friends who also have heart disease. Sometimes I practice at the park with my friends and we all enjoy it. Since I retired, this has been a good opportunity to have something to do with them. This made me more willing to go and practice. (ID: 8)*

### 3.3. Feeling good

The category “Feeling good” provided insight into participants’ perceived effects after practicing *Baduanjin*. Participants reported perceived improvements in their physical and mental health, and they become more active in their daily life.

In terms of physical health, after practicing *Baduanjin* for several weeks, participants described that they had become more active in their daily life as they experienced less shortness of breath. Participants also said that they gained confidence walking longer distances or at faster speeds, or even just had the confidence to get out of the house:

*I used to keep a walking stick in my backpack, and I had to walk on the walking stick at all times. Now, I can walk without it. Sometimes I can walk more than 20,000 steps, and sometimes I will challenge myself to see how far I can go. (ID: 10)*

In addition, participants experienced other improvements in their physiological health. Some participants mentioned gradual improvement in their digestion, and improved appetites following participation. Some participants had suffered intractable constipation for multiple years, but now reported regular bowel movements without constipation.

*I think my digestive system has become stronger and now I have a better appetite. Recently, I have been eating more than I did in the past. Moreover, I was often constipated before, but now I no longer suffer from constipation. (ID: 3)*

Moreover, participants also said that their mental wellbeing states had improved. Participants reported feeling comfortable every time they finished practicing *Baduanjin*. They also felt relaxed, as they said *Baduanjin* calmed them, gave them pleasure, and eased their anxiety. Participants also reported that they had obtained positive energy and vigor from *Baduanjin*. Other perceived benefits were an improvement on their moods (becoming more hopeful) and sleep patterns (bot in extended time and faster getting asleep).

*Every time after I practice Baduanjin, I feel relaxed. I haven’t been so relaxed in a long time. To be honest, after getting this disease, I lost confidence in myself and there was no hope in life. But after a period of exercise, I felt that my mental state was much better and more energetic than before, and my thoughts were not so negative. (ID: 14)*

*I used to be afraid to sleep. Following several weeks of Baduanjin qigong training, I could get to sleep as soon as I lay down. (ID: 3)*

## 4. Discussion

This is the first study to explore CHF patients’ experiences’ using *Baduanjin* as a cardiac rehabilitation regimen. The results provide insight into participants’ learning and practice process, adherence, and perceived effects. While *Baduanjin* postures have a quick learning curve, their proper execution requires time and consistent practice. Coordinating the mind, the movements, and breathing in *Baduanjin* exercise is only possible progressively, through repeated practice. Participants experienced many benefits, for both mental and physical health, with regular high-quality *Baduanjin* practice. Moreover, the study showed that *Baduanjin’s* perceived training benefits were crucial to adherence to home-based training. In addition, internal facilitators such as trust in *Baduanjin* and attitudes toward health, as well as flexibility and external support, are also important for patients to keep practicing.

*Baduanjin* is an aerobic exercise in which the movements are simple, slow and relaxing. With varying skill levels, people may perform one style of *Baduanjin* in different ways. This may lead to variation in physiological responses. A previous study has shown that skill level may exert a sizable effect on metabolic and cardiorespiratory responses to Tai Chi practice (a traditional exercise similar to *Baduanjin*) (40). The authors found a higher mean heart rate and deeper breathing among the high-level practitioners. In our study, many participants experienced difficulty achieving maximum training effects if *Baduanjin* was practiced incorrectly. A high-level practitioner can perform *Baduanjin* with high quality, especially for the handful of movements which are particularly difficult. For example, high-level *Baduanjin* practitioners tend to perform those movements requiring participants to squat with lower positioned technique than the low-level practitioners, thus requiring increased muscle contraction from the lower extremities. Therefore, the improvements in their exercise capacity are expected to be more apparent. As most of the participants in this study were new to *Baduanji*n, several challenges to reaching postural accuracy were identified. These suggested that additional instructions should be added to the teaching course or demonstration videos in clinical practice.

Coordinating the mind, the movements and breathing in *Baduanjin* exercise is only achievable over time, with repeated practice. Unlike other types of exercise, *Baduanjin* involves both “internal” and “external” effort, and in this way, it trains both the body and the mind. Firstly, while practicing *Baduanjin*, the practitioner not only must move their body, but also circulate *qi* (vital energy) throughout the body *via* their breath. While practicing *Baduanjin*, a high-level practitioner should breathe in harmony with the movements in order to circulate *qi* throughout the body. However, a basic-level practitioner would not do it this way, because he or she would only have to concentrate on movement, maintaining natural breath. Secondly, with practice, a novice will gradually be able to concentrate their mind on each movement. However, only by achieving a certain level will they be able to coordinate the breathing and the movements with the guidance of conscious mental effort (10). The importance of the coordination between the mind, the movements and breathing has been reported in a previous qualitative study exploring the mind-body connection during Yoga (41). Hence, this implies the need for more guidance and explanation on the connection between mind, body, and breathing for *Baduanjin* practitioners in future.

With regular practice and rehearsing of the gentle movements, as well as the coordination of breath, *Baduanjin* practitioners may improve their strength, physical fitness, and function of multiple organs and systems. In addition to being accessible and learner-friendly, *Baduanjin*’s eight simple postures are also proven to be suitable in terms of their exercise intensity for CHF patients (23). Moreover, purported mind-body therapy mechanisms may be relevant for CHF pathophysiology, such as targeting various aspects of breathing and relaxation, mitigating sympathetic overdrive, modulating autonomic tone, and addressing the neurohormonal axis (42–44). This finding is consistent with existing literature which indicates that regular *Baduanjin* practice could promote the physical health of CHF patients. For example, *Baduanjin* practice can increase one’s capacity for physical activity and general physical fitness (45), enhance cardiopulmonary function (46), and improve sleep quality (21).

In terms of mental health, *Baduanjin* can relax, calm, and please practitioners’ minds, and therefore improve psychological and mental health. From the perspective of Chinese medicine, *qi* (vital energy) circulating throughout the body is intertwined with both psychological and mental health. Negative psychological states, for example anger, depression, irritability, and restless mood may stagnate *qi*. An underlying philosophy of the practice is that *Baduanjin* cultivates the balance and harmony of *qi* to maintain a healthy body (47). *Baduanjin* focuses on mobilizing functional potentialities, regularizing the breathing process, and unifying the mind and body through the regulation of breathing, thereby promoting normal *qi* circulation (48). Normal *qi* circulation can also regulate passive emotions. Regarding biological mechanisms, it has been reported that the activity and connectivity of key brain regions pertaining to depression, the autonomic nervous system, and neuroinflammatory sensitization are modulated *via* mind-body exercise (49, 50). Similarly, an interview study of 20 community elderly individuals who practiced *Baduanjin* for 40 min every day for 12 weeks reiterated that they felt psychologically relaxed, due to the self-tranquility, the pleasure, and the relief from emotional symptoms (51).

Our findings regarding intrinsic motivation to do *Baduanjin* exercise provide insight into the high adherence in this sample. “Intrinsic motivation” refers to the inherent satisfaction which individuals derive from physical activity. This includes feelings of enjoyment and accomplishment (52). Intrinsic motivation has been defined as the inherent satisfaction that drives one to undertake a particular activity (52). Motivation is a critical factor underlying sustained exercise. In turn, it is also associated with important health outcomes. In the interview, adherent participants often referenced their improvements as walking further, and improved their moods through participation in *Baduanjin* training. Therefore, it appears as if both actual and perceived improvements in health may serve as key mechanisms of long-term exercise adherence. This echoes the findings of a qualitative study regarding exercise adherence in patients with CHF (53) which reported that patients with CHF tended to be motivated by improved health and performance in their daily activities (53). Thus, when working with CHF patients starting an exercise program, highlighting improvements can support long-term adherence to exercise. Our findings also demonstrate the importance of autonomous regulation in fostering physical activity. Adherent participants mentioned that whether to exercise was one’s own choice, and that this was primarily decided by the degree to which they cared for their own bodies. This underscores the need to examine the goals and self-regulatory features associated with regular participation in exercise and other forms of physical activity.

Good adherence was also attributed to *Baduanjin’s* in-home setting. Home-based modalities might be more practical options for increasing patients’ accessibility to EBCR. This is because a third of Chinese CHF patients have been found to either rarely leave the home or are unable to do so due to their symptoms (54). Home-based exercises also empower patients to take personal responsibility and accountability for their disease management (55). Moreover, environmental constraints are mitigated, particularly the challenge of limited healthcare resources, including insufficient rehabilitation facilities, a paucity of medical insurance, and poor access to hospital services in many of China’s rural areas (56). According to a recent consensus statement on EBCR delivery in low-resource settings, safe, equipment-free, cheap, and easy-to-implement exercise modalities provide the most practical options for implementation in China (57).

This study has several strengths. We took several steps to enhance the findings’ credibility. To achieve dependability, we read the interviews several times to derive a general understanding, and there was also frequent comparison between the parts of the analysis and the complete interview texts. All interviews were conducted by one researcher (HZ), which also added to the results’ dependability and she could use experiences from earlier interviews in probing for more information. To achieve confirmability, several researchers with different backgrounds were involved in the analysis process and discussed the findings. This minimized the risk of inventing data or biased interpretation. As this qualitative study was nested in the pilot RCT, current qualitative study should help to explain, support, and complement the findings from the pilot RCT.

Regardless, there remain limitations to this study. Given that this qualitative study was nested in a pilot RCT with a small sample (*n* = 20), variation in patients’ characteristics were limited to a sub-set with heart failure. For example, all of the study population was male and relatively young; and no patients were included in the study with NYHA I or VI. Although we encouraged participants to elaborate on their perspectives on the research topic, it is possible that female, younger or elderly patients with HF could have different perceptions on *Baduanjin*. Yet, we still contend that the main findings described above are generally transferrable to other regions of China. This is because that the study participants’ demographic and clinical characteristics are similar to the those CHF patients who are undergoing a cross-sectional survey in Guangzhou (58), and in China in general (54). Moreover, *Baduanjin* is commonly accepted as being beneficial to one’s health and relatively easy to learn in a short time, it has been a community exercise throughout different regions of Mainland China (59), Taiwan, Macau, and Hong Kong (30, 60). Hence, similar experience of practicing *Baduanjin* are expected to be found. Moreover, future research should include more participants receiving *Baduanjin* intervention under the real world treatment environment.

## 5. Conclusion

Positive practicing experience and perceived training benefits from *Baduanjin* was reported among the participants, which indicates that *Baduanjin* has the potential to become a cardiac rehabilitation exercise modality for CHF patients in China, especially in a home-based setting.

## Data availability statement

The raw data supporting the conclusions of this article will be made available by the authors, without undue reservation.

## Ethics statement

The studies involving human participants were reviewed and approved by the Ethics Committee at the Guangdong Provincial Hospital of Chinese Medicine (number: B2016-202-01). The patients/participants provided their written informed consent to participate in this study.

## Author contributions

XkC, WJ, ZW, XxC, and CL contributed to the conception and design of the research. ZW and XxC contributed to obtaining funding. XkC and HZ contributed to data collection and data analysis. XkC, WJ, HZ, and MS contributed to interpretation of the results. XkC and WJ drafted the first version of the manuscript and revised it based on other authors contribution. All authors contributed important intellectual content to the critical revision of the manuscript and read and approved the final manuscript.
